# Acute and chronic effects of local muscle vibration training inducing illusions on wrist strength and neurophysiological measures

**DOI:** 10.1038/s41598-025-26915-z

**Published:** 2025-11-28

**Authors:** Sophie Julliand, Jérémie Gaveau, Alain Martin, Nicolas Amiez, Adrien Guzzo, Martine Lemesle-Martin, Davy Laroche, Charalambos Papaxanthis

**Affiliations:** 1 INSERM, CIC 1432, CHU Dijon-Bourgogne, Centre d’Investigation Clinique, Module Plurithématique, Plateforme d’Investigation Technologique, 21000 Dijon, France; 2 INSERM U1093-CAPS, UFR des Sciences du Sport, Université Bourgogne Europe, 21000 Dijon, France; 3 Neurophysiologie Clinique, CHU Dijon, Service Hospitalo-Universitaire, 21000 Dijon, France

**Keywords:** Vibration, Illusion, Excitability, Intracortical inhibition, Strength, EEG, Neuroscience, Motor control

## Abstract

Local muscle vibrations (LMV) acutely and chronically affect the sensorimotor system, but their effects vary depending on application conditions. The mechanisms behind repeated LMV exposure are still debated. This study investigated the chronic effects of LMV under illusion-inducing conditions on the upper limb. Nineteen healthy participants completed 9 LMV sessions (80 Hz, 20 min) over 11 days targeting the right wrist flexors. Illusions were assessed using subjective scales and EEG (alpha band desynchronization). Neurophysiological parameters (spinal, corticospinal excitabilities, and intracortical inhibition) and grip strength were evaluated before and after a control session (20-min rest) and an LMV session, as well as after 9 LMV sessions and 5 days post-protocol. Participants consistently experienced illusions throughout the protocol, with stronger perceptions during the first 15 min. However, subjective and objective measures were found to be independent. Acute LMV significantly reduced the H-reflex (-52.3% [-69.7 ; -21.0]) but did not affect strength or other neurophysiological measures. Additionally, repeated LMV exposure induced no chronic changes in strength or neural excitabilities and inhibition. While confirming acute LMV effects under illusion conditions, this study found no evidence of chronic adaptations. It also suggests that subjective and objective (EEG-based) illusion measures reflect distinct neurophysiological mechanisms.

**Supplementary Information:**

The online version contains supplementary material available at 10.1038/s41598-025-26915-z.

## Introduction

Local Muscle Vibrations (LMV) are a non-invasive neuromodulation technique that effectively stimulates local sensory receptors. Since the 1970s, LMV have been widely used to explore the sensorimotor system^1–4^. More recently, their potential therapeutic applications have gained attention, particularly in neurological rehabilitation^5,6^. Despite this growing interest, the mechanisms underlying the LMV-induced modulations remain only partially understood, particularly in two key contexts: when LMV induce movement illusions, and when applied repeatedly over time. To address these gaps, we aimed to evaluate both the acute (single session) and chronic (repeated sessions) adaptations of LMV on the neuromuscular system under conditions known to induce movement illusions.

In healthy participants, an acute application of LMV produces temporary neuromuscular modifications at the vibrated site, mainly including transient changes in muscle strength, intrinsic motoneuronal excitability, and a significant reduction in spinal excitability as reflected by a decrease in Hoffmann’s reflex (H-reflex)^4,7–10^. In addition to spinal adaptations, acute enhancement of cortical excitability has been suggested following LMV. Evidence from neurophysiological studies, which compared motor evoked potentials (MEPs) with cervicomedullary (CMEPs) or thoracic motor evoked potentials (TMEPs), has demonstrated increased MEP/CMEP or MEP/TMEP ratios following LMV^7,11,12^. While MEP reflects corticospinal excitability, CMEP and TMEP serve as indicators of motoneuronal excitability. Findings from electroencephalography (EEG) further support these observations, as they reveal strong desynchronization in the alpha power spectrum over the contralateral sensorimotor cortex (electrodes C3 or C4), indicating increased cortical activity during LMV that may persist post-vibration^13,14^. While some research partly attributes these findings to changes in intracortical excitability, such as reduced intracortical inhibition and increased facilitation^15,16^, the results remain inconsistent, with other studies reporting no significant effects on these two parameters^17^.

The variability of LMV effects likely stems from differences in application parameters^4,10,18–20^. A particularly relevant distinction is whether movement illusions are evoked during LMV. For example, when LMV is carefully applied to a relaxed and hidden limb, with frequencies ranging from 70 to 110 Hz and a small amplitude (< 3 mm)^20^, the literature consistently reports that LMV can create the sensation of movement illusions, giving the impression that the vibrated muscle is being stretched^22^. These illusions arise from the heightened activation of muscle spindles by LMV, which generate robust proprioceptive signals via Ia afferent fibers that project towards the spinal cord up to the brain^23^. Research has shown that LMV-inducing movement illusions elicit greater cortical excitability compared to LMV without illusions. For instance, functional MRI studies have demonstrated significant activation of the premotor, sensorimotor, and parietal cortices during LMV, with greater activation when movement illusions are present^24,25^. Interestingly, these cortical regions overlap with those activated during real movements^26^, suggesting that movement illusions engage comparable neural structures as real movements, albeit to a lesser extent. EEG studies have reported corroborating findings, showing a strong modification of activity in the alpha or Mu band (8–13 Hz) during LMV, which becomes even more pronounced when movement illusions occur^27,28^. Recent work by Amiez et al. (2024a)^18^ compared two LMV conditions, both applied to a relaxed limb: one inducing movement illusions and the other without illusions but with Tonic Vibratory Reflex (TVR). Their findings revealed distinct acute after-effects on muscle strength; specifically, the vibrated muscle experienced a greater strength reduction in the TVR condition. These results highlight the importance of precisely controlling LMV parameters, as the presence or absence of movement illusions can differentially modulate neurophysiological and functional responses following LMV.

While LMV are increasingly used in clinical rehabilitation, often applied multiple times per week over several weeks^29,30^, studies examining their cumulative effects (repeated applications) remain limited, and even less is known about how repeated illusion-inducing LMV might shape neuromuscular adaptations. Such research is critical to understanding the underlying neural mechanisms and optimizing their application. Preliminary evidence suggests that acute and chronic adaptations of LMV may differ^4,19,31–34^, further emphasizing the need for controlled investigations. Notably, the effect of repeated LMV sessions under conditions that induce movement illusions has not yet been systematically explored.

This study aimed to investigate both the acute (after a single session) and chronic (following repeated sessions) adaptations of LMV induced illusions on wrist flexors. We assessed movement illusions using subjective scales and EEG measures. Key neurophysiological outcomes included corticospinal excitability (MEPs), spinal excitability (H-reflex), intracortical inhibition (SICI), and functional performance (maximal grip strength). These parameters were measured at various time points throughout the LMV protocol. Based on prior findings, we hypothesized that a single session would not significantly reduce strength under illusion conditions^18^, while repeated sessions would produce distinct chronic effects on grip strength (i.e., no change) compared to previous LMV chronic protocols without illusions^33,34^.

## Methods

### Participants

Nineteen (*n* = 19) volunteers were included in this protocol (9 females and 10 males, mean age 26.6 ± 5.3 years). All participants were right-handed (mean score 0.8 ± 0.2) as determined by the Edinburgh Handedness Inventory^35^. None of the volunteers had any neurological, psychological, or musculoskeletal diseases. The protocol was approved by CPP Ile de France IV (number 2021-A03219-32, and ClinicalTrials NCT05315726), and all participants provided verbal informed consent, in accordance with the revised 2013 Declaration of Helsinki.

## General experimental setup

**Fig. 1:**
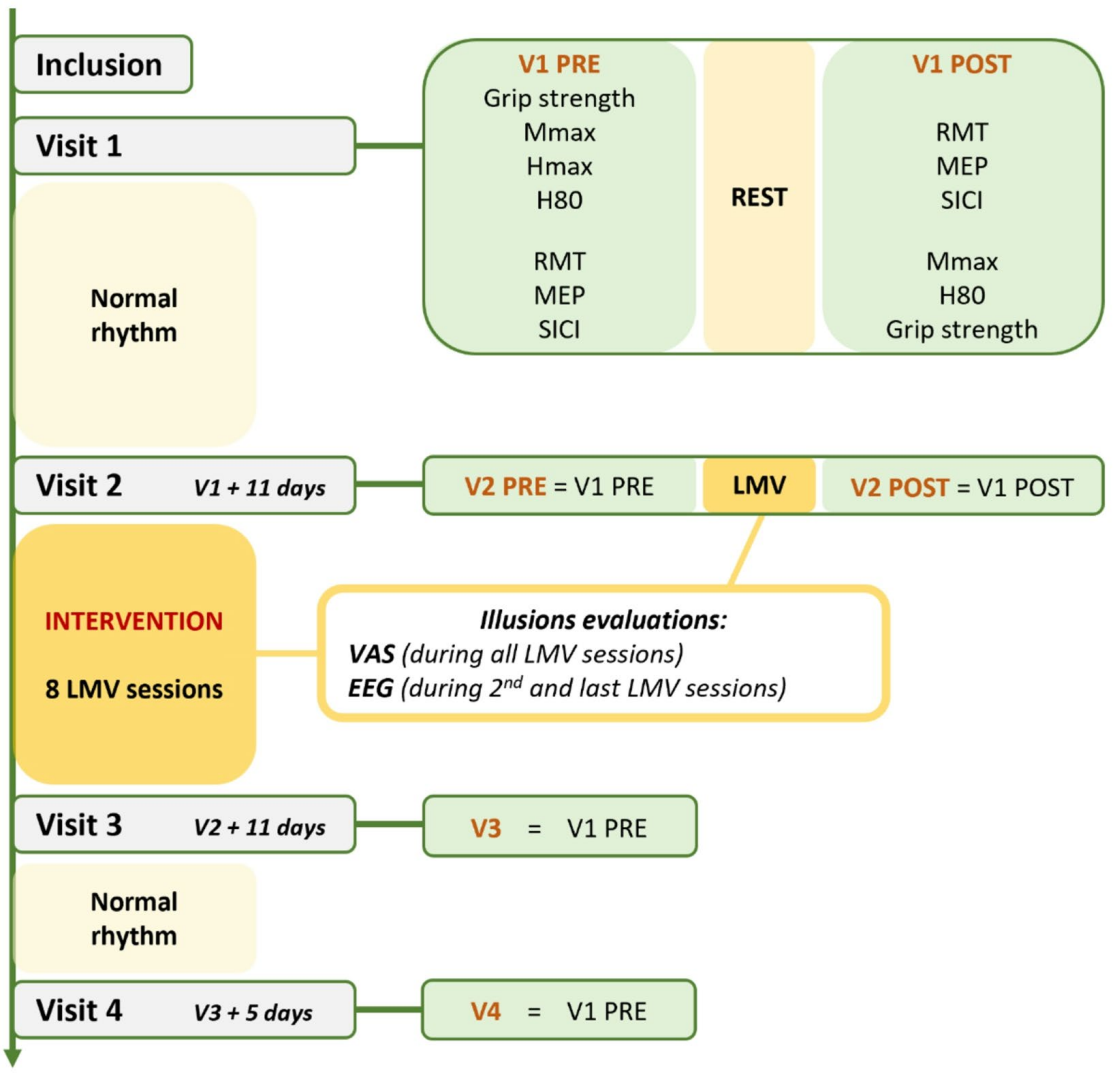
Study design. Neuromuscular evaluations were conducted during four visits, V1 to V4. V1 served as a control condition for V2. V2 assessed the acute effects of LMV, while V3 and V4 evaluated their chronic effects. All neurophysiological evaluations were conducted on a relaxed right flexor carpi radialis (FCR) muscle under identical conditions. The intervention consisted of 9 vibratory sessions on the right wrist flexors, starting with the first session at V2 and ending with the final session the day before V3. The LMV protocol aimed to optimize movement illusions. Illusions were quantified using EEG (during the second and last LMV sessions) and subjective scales during all LMV sessions. Maximal M-wave (Mmax); Maximal Hoffman reflex (Hmax); 80% of Hmax (H80); Resting Motor Threshold (RMT); Motor evoked potential (MEP); Short intracortical inhibition (SICI); Electroencephalogram (EEG).

Visits 1 (V1) and 2 (V2) each included two measurement blocks (PRE and POST, see Fig. 1), separated by a 20-minute rest at V1 and a 20-minute LMV of the wrist flexors at V2. V1 and V2 were 11 days apart, during which participants were instructed to maintain their normal lifestyle. V1_PRE_, V1_POST,_ and V2_PRE_ were used to assess data reproducibility over time and served as control conditions.

The LMV protocol started after V2_PRE_ and finished the day before V3, with participants receiving a total of nine 20-minute LMV sessions. Movement illusions were evaluated subjectively (via ratings) in all sessions and objectively (via EEG) during the second (EEG1) and final (EEG2) sessions, due to the demanding nature of EEG recordings.

In V3 and V4, the same neuromuscular measurements as in V1_PRE_ and V2_PRE_ were recorded. Between V3 and V4 (5 days), participants maintained their normal lifestyle. The acute effects of LMV on inducing illusions were assessed by comparing neurophysiological parameters and strength using V1_PRE_, V1_POST_, V2_PRE,_ and V2_POST_. The chronic effects of repeated LMV with illusions were assessed by comparing V2_PRE_ with V3 and V4, with V4 capturing any lasting changes, as previously shown in the literature, up to 2 weeks following the completion of the LMV protocol^4,34,36^.

All visits (V1-V4) were consistently scheduled at the same time of day for each participant to minimize potential intra-participant circadian fluctuations^37,38^, and followed a consistent weekly schedule: V1 (Thursday), V2 (Monday), V3 (Friday) and V4 (Wednesday).

**Fig. 2:**
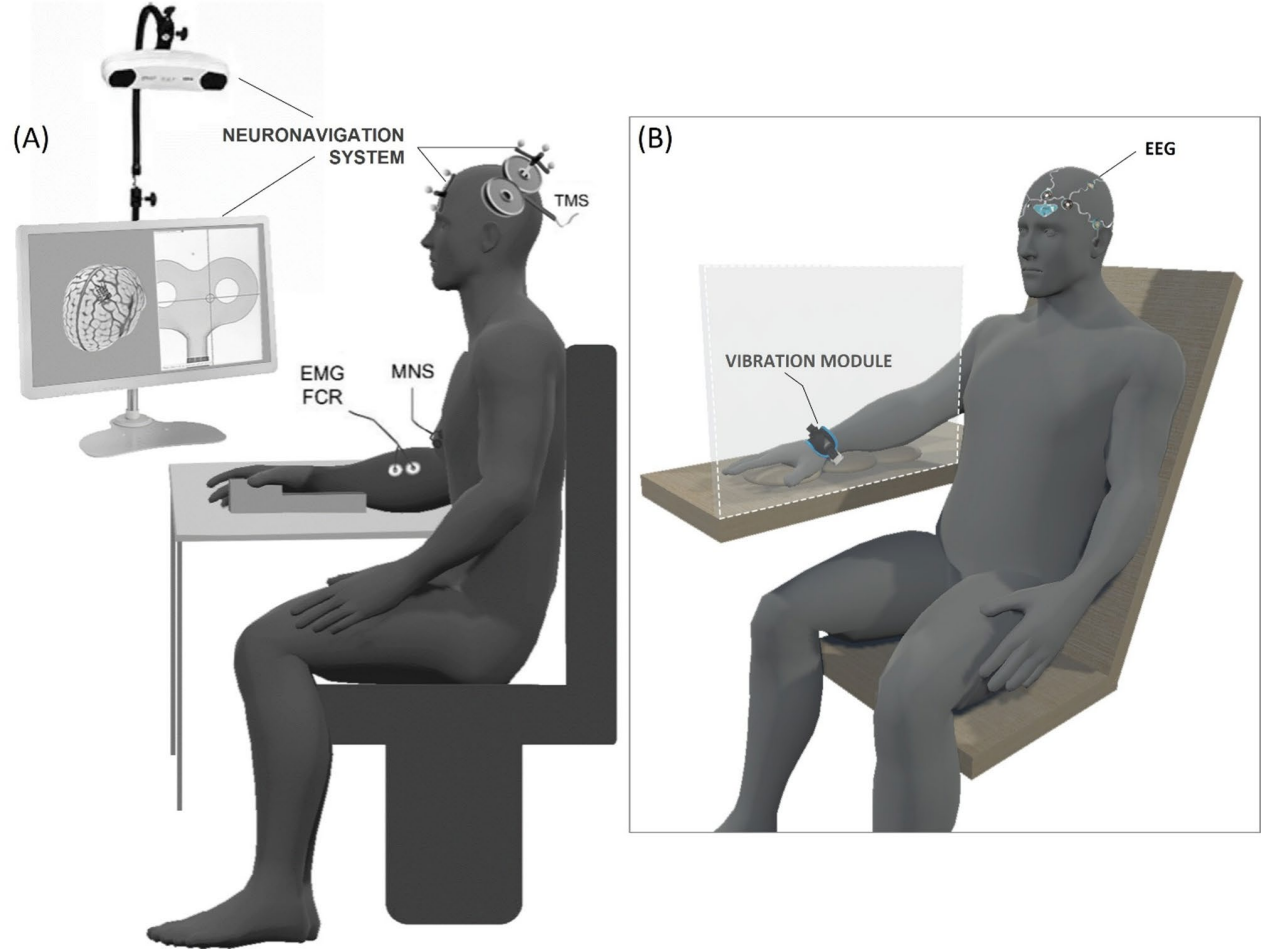
Schematic view of the experimental setup and participant positions. (**A**) The measurements recorded in the Flexor Carpi Radialis (FCR) using a Neuronavigation system, and with Transcranial Magnetic Stimulation (TMS), Median Nerve Stimulation (MNS), and Electromyography (EMG) devices. (**B**) The position of the vibratory device. Additionally, an Electroencephalogram (EEG) was performed twice, during the second and last LMV sessions.

During the neurophysiological measurements (Fig. 2A), participants were comfortably seated upright in a chair, with their right forearm supported in a semi-pronated position on a custom-made support, elbow at 90° flexion, and shoulder at 30° abduction, 15° flexion, and 20° external rotation. During the 20-minute rest (V1) and all LMV sessions (Fig. 2B), they sat in a semi-reclined position to promote muscle relaxation and enhance the likelihood of experiencing illusions^20^. Their arm was hidden from view and supported at the elbow and wrist by malleable cushions, with the elbow semi-flexed, pronated, and the wrist in a neutral position.

### Vibrations

Participants completed nine vibratory sessions, one each day over two consecutive weeks, each lasting 20 min of continuous mechanical LMV. The vibration module (Vibramoov, Technoconcept, Manosque, France) was directly applied to the skin, approximately 2 cm proximal to the right wrist crease, and secured around the forearm with a strap (Fig. 2B). To ensure optimal comfort and appropriate pressure, the wristband size was individually adjusted for each participant. Continuous LMV was administered for 20 min at a frequency of 80 Hz with an amplitude of 2 mm (according to the manufacturer’s specifications). The participants’ view of their forearm was obstructed with a cardboard box to induce movement illusions and diminish the tonic vibratory reflex^20,39^. Participants were instructed to relax and concentrate on the LMV. The 20-minute duration was chosen based on previous studies demonstrating its effectiveness in inducing movement illusions and in modulating H-reflex and muscle strength^7,10,18^. In particular, we drew on the findings of Nito et al. (2021)^10^, who reported that corticospinal excitability of the vibrated muscle (FCR) was already significantly modulated after as little as 8 min of LMV.

### Illusions

During each vibratory session, three visual analogue scales were used to rate participants’ subjective sensations. These scales, ranging from 0 (non-existent) to 10 (very strong), assessed the intensity of the illusory movement (strength), the duration of the illusion during the last minute (continuity), and the clarity of illusions as if a real wrist extension was performed (vividness)^24^. Participants were asked to evaluate their sensations at specific intervals (1 min, 5 min, 10 min, 15 min, and 19 min), providing one score per scale at each time point. The pooled score, calculated as the mean of the three scales, served as the marker for the overall intensity of the illusions.

Two EEG sessions were conducted during the second and final vibratory session (i.e., EEG1, and EEG2, see Fig. 1). EEG signals were recorded (Icecaps, Neuronaute, BioSerenity, Paris, France) from 21 electrodes, based on the 10–20 system. Electrode impedances were maintained below 5 kω, and signals were recorded at a sampling rate of 500 Hz. The entire EEG assessment was performed with participants’ eyes closed. Two 5-minute rest periods were recorded at the beginning and end of the 20 min of LMV to assess the resting state (REST PRE and REST POST). An accelerometer, positioned on the vibrator module and connected to the EEG system, allowed for the detection of when vibrations were on/off for synchronization with the EEG recording.

Research indicates that LMV applied to a resting limb enhances cortical activity, with Mu rhythm ERSP at C3 serving as a marker for this activation^27,28^. Movement illusions induced by LMV further amplify this cortical activity^24,27^. Since our LMV conditions were optimized to induce these illusions, Mu desynchronization during the 20-minute LMV, in comparison to the resting period, was used in all participants to quantify brain activity under illusions.

### Neurophysiological and strength measurements

#### EMG recordings

After shaving and dry-cleaning the skin with alcohol, silver chloride surface electrodes (Ag/AgCl) were positioned on the belly of the right *Flexor Carpi Radialis* (FCR) with an interelectrode distance of 2 cm (center to center). To ensure consistent electrode placements across visits, the distance from the medial humeral epicondyle and the midpoint of the distal wrist crease was measured. Electrodes were positioned at 35% of this distance from the proximal reference point (participants’ mean was 9.82 ± 0.83 cm), corresponding to the theoretical optimal electrode site^40^. A reference electrode was placed over the medial epicondyle of the radial styloid. To prevent noise during LMV, the ground electrode was placed on the olecranon process. The EMG signal was amplified (gain 1000) and band-pass filtered (10–500 Hz), digitized at a sampling rate of 2000 Hz, and recorded for off-line analysis (Biopac System Inc., Goleta, CA, USA).

Neuromuscular tests were conducted in a fixed sequence (see Fig. 1) to minimize potential external influences, aside from rest or LMV, on the primary outcome measure, short-interval intracortical inhibition (SICI). SICI assessments were performed last during the pre-test sessions and first during the post-test sessions. This order was designed to respect critical methodological dependencies (e.g., Mmax preceding H-reflex, RMT before MEP and SICI), and to prevent any active muscle contractions from impacting the neurophysiological parameters of interest^41^.

#### Maximum isometric grip strength

A warm-up phase consisting of 2 min of right wrist and finger flexions starting from low to submaximal intensity preceded the maximum force evaluation. Three (*n* = 3) maximal grip voluntary contractions (T.K.K.5401 GRIP-D hand-grip dynamometer, Takei Scientific Instruments Co., Ltd, Tokyo, Japan), each lasting 3 s with 30 s recovery in between, were performed in each PRE (V1-V4) and POST (V1 & V2) visits (Fig. 1). The participants’ positions were similar across sessions, with their elbows and forearms resting on their right thigh while squeezing the dynamometer. Verbal encouragement was provided during every contraction. The maximal grip strength for each set of contractions was determined as the highest value among the three trials, displayed on the dynamometer screen.

#### Peripheral electrical stimulation

The H-reflex and M-wave at the right FCR were elicited by stimulating the right median nerve. Rectangular 1 ms wave pulses were delivered through bipolar felt pad electrodes, which were secured around the arm using a Velcro band, and connected to a constant-current stimulator (DS7R, Digitimer, Welwyn Garden City, Hertfordshire, UK). The optimal stimulation site, determined on each visit as the location that produced the largest M-wave, was identified near the medial border of the cubital fossa, approximately 2 cm above the medial epicondyle of the humerus. In each session, the stimulation intensity was incrementally increased until the largest H-reflex and the M-wave reached a plateau. Three stimulations were conducted at 120% of the plateau intensity, and the M-wave with the greatest peak-to-peak amplitude was designated as Mmax (mean intensity across visits: 16.0 ± 6.3 mA).

For the H-reflex, the initial visit (V1_PRE_) was used to determine a reference value. Three stimulations were performed at the intensity eliciting the largest H-reflex; the average of these three peak-to-peak amplitudes was used to calculate Hmax. Subsequently, 10 stimulations were delivered at the intensity evoking 80% of Hmax (H80) within the ascending part of the recruitment curve. During the following PRE and POST visits, to ensure consistent stimulus conditions for evaluating the H80 across these different time points, a stable M-wave was maintained, corresponding to the M-wave associated with H80 (M(H80))^42^; the mean intensity across visits was 6.9 ± 3.4 mA.

#### Transcranial magnetic stimulation (TMS)

TMS was applied to the left primary motor cortex using a 70 mm figure-of-eight coil connected to a monophasic Magstim BiStim^2^ stimulator (The Magstim Co., Whitland, UK). The hot spot, defined as the optimal stimulation site producing the largest Motor Evoked Potential (MEP) amplitude for a given intensity, was determined with the assistance of a navigated brain stimulation system (Brainsight TMS Navigation, Brainbox, Cardiff, UK). The hot spot, identified as one of the 12 points on the system map (mean MNI coordinates: -44.7, 13.8, 85.7; Négyesi et al., 2020^43^), was determined individually for each participant during V1 and remained consistent for that participant across all visits. The resting motor threshold (RMT) was defined as the minimum stimulus intensity required to evoke at least 5/10 MEPs with a peak-to-peak amplitude of 50 µV in the relaxed FCR^44^. A block of 15 single-pulse stimuli was delivered at 130% of RMT to measure MEP. Another block of 15 paired-pulse stimulations at 80%-130% of RMT with 3-ms intervals was carried out to study Short Intracortical Inhibition (SICI)^45^.

### Data analysis

#### Processing

A tailored MATLAB algorithm (MATLAB R2024a; MathWorks, Natick, MA) was developed to extract data from each stimulation (V1-V4), including peak-to-peak amplitudes of Hmax, M(Hmax), H80, M(H80), Mmax, MEP, and SICI. Normalization (% expression) of Hmax, M(Hmax), H(80), M(H80), and MEP was performed relative to Mmax, enabling intra-sessions and interindividual comparisons^42,46^.

SICI was computed with the following formulae^46^:

$$\:SICI\%\left(Mmax\right)=\frac{(MEPcond-MEPtest)}{Mmax}\:\times\:100$$

*MEP*_*cond*_ *is a MEP evoked at 130% of the RMT preceded 3 ms before by a stimulation at 80% RMT. MEPtest is a MEP evoked at 130% of the RMT.*

Negative values signify inhibition, while positive values indicate facilitation.

The interquartile range (IQR) method was employed to detect outlier values within each participant, stimulation block, and session (see Supplemental Table A.1). An outlier was identified if the value exceeded Q3 + 1.5 x IQR or was less than Q1–1.5 x IQR^7,47^. Due to the limited dataset (i.e., only 3 values), this method was not applied to Mmax, Hmax, and M(Hmax).

During LMV, some participants exhibited a small tonic vibratory reflex (TVR)^3,48^. Complementary EMG analysis, similar to the methodology used by Amiez et al. (2024a)^18^ was carried out. The results revealed an average TVR of 0.08 ± 0.05%Mmax, ranging from 0.02% to 0.17% of Mmax (averaged from V2 and V3). These findings align with those reported in the illusions group of Amiez et al. (2024a)^18^ (0.05 ± 0.03%Mmax) compared to 0.22 ± 0.18%Mmax in their TVR group. Additionally, our EMG analysis during maximal grip strength closely matches their results, as our participants demonstrated an average of 6.01 ± 3.97%Mmax, comparable to the ~ 6% reported in their study.

#### EEG

EEG processing and calculations were performed using the MNE Python library^49^. EEG data were filtered with a 0.5 to 40 Hz band-pass filter. To improve Independent Component Analysis (ICA) decomposition, the initial 180 s of acquisition were excluded to eliminate noise caused by eye movements or other disruptions typically occurring during the transition to a resting state. ICA decomposition was performed, and components related to eye movements and muscle artefacts were manually removed.

ERSP Analysis Event-Related Spectral Perturbations (ERSPs) were calculated using Morlet wavelets with a cycle length that progressively increased from 1 to 30, spanning a frequency range of 1 to 30 Hz across the full experiment duration^49^. An 80-second baseline was applied during the resting state (-110 to -30 s). For analysis, only the mu band (8–14 Hz) was extracted from the ERSP data.

### Statistical analysis

#### Sample size

A power analysis was conducted using G*Power (version 3.1.9.4) to determine the required sample size for this protocol. The calculation was based on previous studies from Souron et al. (2018, 2017)^33,34^, which examined chronic strength differences following LMV training. Assuming a medium effect size (f = 0.32), an alpha level of 5%, a statistical power of 90%, and one group with four repeated measurements, the analysis indicated that 19 participants were needed.

Statistical analyses were conducted using JASP (version 0.19.0.0 ; University of Amsterdam, The Netherlands), and “rmcorr R package” for repeated-measures correlations^50^. Data normality was verified using the Shapiro-Wilk test.

#### Validation of the SICI protocol

One-sample t-tests (comparing with the zero ‘0’ value) were performed to confirm that the protocol successfully induced SICI during the control conditions (V1_PRE_, V1_POST_, and V2_PRE_), as described and illustrated in the supplemental Figure A.1.

#### Effects of LMV on illusions

To verify that our LMV protocol successfully induced sensory illusions, we calculated the average pooled score from the three visual analogue scales over the 20-minute session and compared this score to the zero ‘0’ value (one-sample t-tests), for the second LMV session (EEG1).

We computed an ERSP_REST_ parameter, which represents the ERSP_MU_ normalized by the baseline ERSP_MU_ (during REST PRE) for each participant.

Then, to evaluate the acute effect of LMV on illusion, we tested the within-session evolution of sensory illusion during LMV (i.e., during one session, EEG1), measured both subjectively (pooled score) and objectively (ERSP_MU_). Two Friedman tests were performed with a factor *Time*: 1’, 5’, 10’, 15’, 19’, for pooled scores; and REST PRE, 1’, 5’, 10’, 15’, 19’, REST POST, for ERSP_MU_. The relationship between subjective (pooled scores) and objective illusion (ERSP_REST_) measures during EEG2 was further analyzed through a Spearman’s correlation for inter-participants and a repeated measures correlation for intra-participant consistency^50^.

Lastly, to examine the progression of illusions across multiple LMV sessions, both intra- and inter-session changes were analyzed. For subjective pooled scores, a repeated measure ANOVA was used with a *Time* effect (1’, 5’, 10’, 15’, 19’), and a Friedman test for the *Session* effect (Sessions 1, 2, 3, 4, 5, 6, 7, 8, 9). For the ERSP_MU_ variable, a Friedman test was used with *Time* (1’, 5’, 10’, 15’, 19’) as a factor. The session effect (EEG1, EEG2) was further analyzed using a Wilcoxon test. Finally, the relationship between subjective (pooled scores) and objective illusion (ERSP_REST_) measures during the last LMV session (EEG2) was analyzed through a Spearman’s correlation for inter-participants and repeated measures correlation for intra-participant consistency.

#### Effects on grip strength and neurophysiological parameters

The acute effects of LMV were assessed using two-factor repeated measures ANOVA for normally distributed variables (RMT and M(H80)), with *Visits* (V1, V2) and *PrePost* (PRE, POST) as factors. For non-normally distributed data (SICI, MEP, Mmax, H80, and Grip strength), a Friedman test was performed with a single factor, *Visits* (V1_PRE_, V1_POST_, V2_PRE_, and V2_POST_). This analysis was followed by a Wilcoxon test to compare the relative changes between PRE and POST in V1 and V2, calculated as follows: V1_RATIO_ = (V1_POST_ – V1_PRE_) / V1_PRE_, and V2_RATIO_ = (V2_POST_ – V2_PRE_) / V2_PRE_.

To evaluate the chronic effects of LMV, a repeated measures ANOVA was used for normally distributed variables (RMT, M(H80), and Grip strength) across the factor *Visits* (V2_PRE_, V3, V4). For data that were not normally distributed (SICI, MEP, Mmax, and H80), a Friedman test was used with the same factor.

#### Effect size, post-hoc analyses, and significance threshold

For ANOVA tests, violations of the sphericity assumption were corrected using the Greenhouse-Geisser adjustment when necessary.

Effect sizes were calculated as follows: For ANOVA, partial Eta squared (ղ_p_^2^) was reported and interpreted as small (< 0.01), medium (< 0.06), and large (≥ 0.14). For the Friedman test, effect size was determined using Kendall’s W (W), categorized as small (< 0.3), medium (< 0.5), and large (≥ 0.5). For the Wilcoxon test, the rank-biserial correlation (r_B_) was computed, following the same interpretation as Kendall’s W.

Post-hoc analyses were applied to all significant results: For ANOVA, pairwise comparisons were corrected using the Holm–Bonferroni method; For Friedman tests, Conover’s post-hoc tests with Holm correction were performed. Exploratory correlations (Spearman) reported in the Supplementary Material (Tables A.3 and A.5) were also corrected using Holm–Bonferroni. Omnibus tests (ANOVA and Friedman) corresponding to our a priori hypotheses were reported without correction. Statistical significance was set at *p* ≤ 0.05 for all analyses.

For all data, normally distributed variables are reported in the text as mean ± standard deviation (SD), while non-normally distributed variables are reported as median with first and third quartiles [Q1 ; Q3].

## Results

### Acute impact of LMV (intra-visit effects)

#### Assessment of protocol efficacy: subjective scales of sensory illusions

Table 1 shows the median [Q1 ; Q3] of the three visual analogue scales used by participants (*n* = 19) to subjectively rate their sensory illusions, which were recorded at specific time points through the 20-minute LMV (i.e., 1, 5, 10, 15, and 19 min) in the session EEG1 (i.e., the day of the first EEG recording). Most participants developed sensory illusions, except for two participants. The median scores during the 20 min were: 4.0 [2.3 ; 5.2] for strength, 5.4 [3.2 ; 7.0] for continuity, and 3.2 [2.5 ; 5.8] for vividness. A one-sample t-test (comparing with the zero ‘0’ value) on pooled sensory illusion values showed significant differences (t = 8.25, *p* < 0.001). The Friedman test did not show a significant *Time* effect (χ^2^ = 2.142, *p* = 0.710, W = 0.028) on the evolution of the pooled illusions over time during the session EEG1.

**Table 1:** Evolution of the three visual analogue scales used to subjectively rate the illusions perceived by participants (*n* = 19), at specific time points during the 20-minute LMV (i.e., 1, 5, 10, 15, 19 min), during EEG1 (on the same day as the first EEG recording).

		Strength	Continuity	Vividness	Pooled
Visit 2 + 1 day (EEG1)	1’	3.0 [0.5 ; 5.5]	6.0 [1.0 ; 7.0]	4.0 [0.5 ; 6.0]	4.0 [0.0 ; 6.0]
	5’	3.0 [2.5 ; 6.5]	5.0 [3.0 ; 8.0]	3.0 [2.0 ; 5.5]	4.0 [2.0 ; 7.0]
	10’	4.0 [2.5 ; 6.0]	7.0 [3.5 ; 9.0]	4.0 [2.0 ; 7.0]	4.0 [2.0 ; 8.0]
	15’	3.0 [2.0 ; 5.5]	6.0 [2.0 ; 8.5]	4.0 [2.0 ; 5.5]	4.0 [2.0 ; 7.0]
	19’	3.0 [2.0 ; 4.0]	5.0 [2.5 ; 8.0]	4.0 [1.0 ; 5.5]	4.0 [2.0 ; 6.0]

### Assessment of protocol efficacy: EEG analysis during LMV

**Fig. 3:**
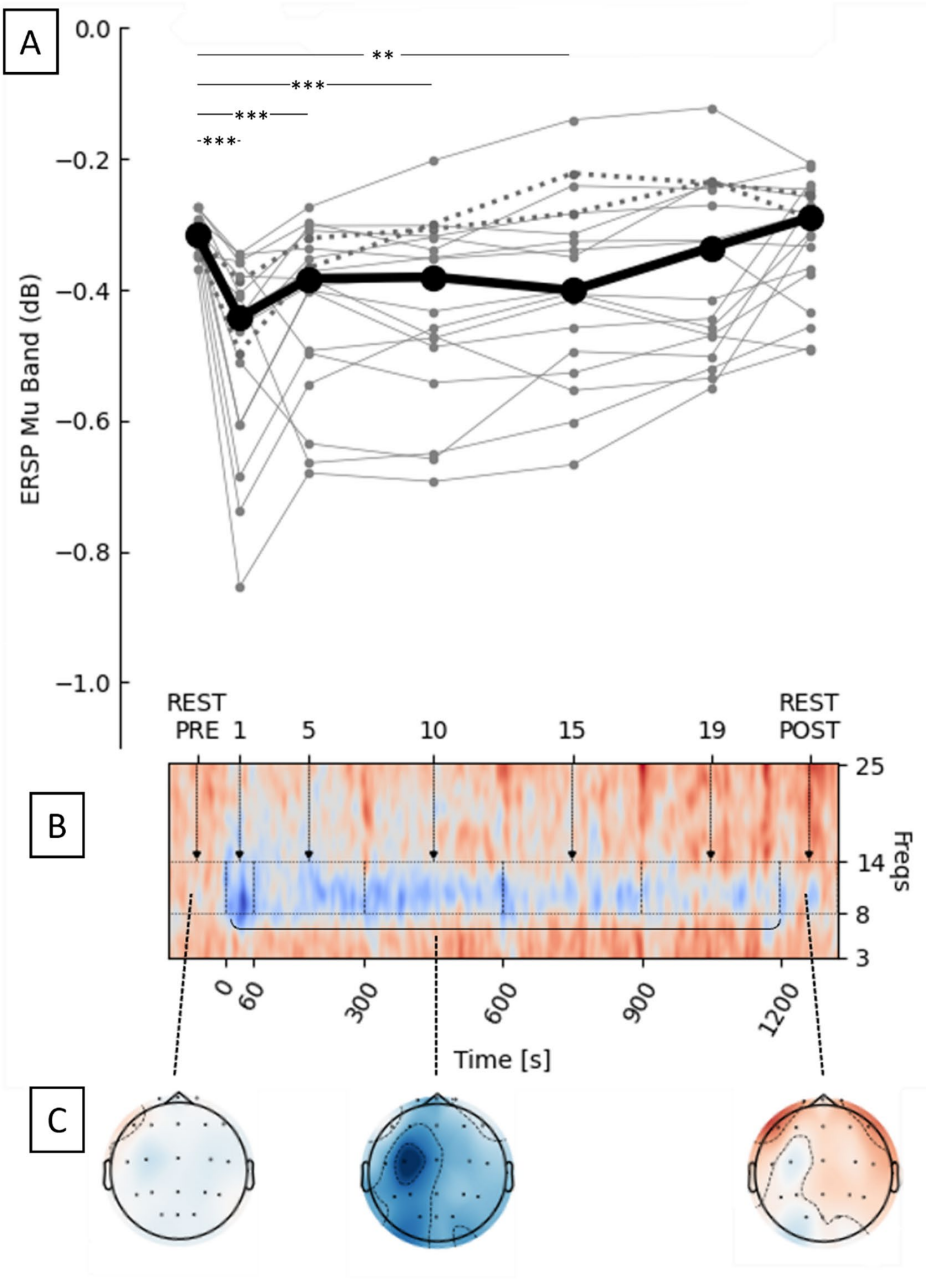
Illustrations of the alpha band evolution before (REST PRE, from − 120s to 0s), during, and after LMV (REST POST from 1201s to 1440s) in EEG1. (**A**) The ERSP_MU_ at different time points: REST PRE, 0–60 s (1), 61–300 s (5), 301–600 (10), 601–900 s (15), 901–1200 s (19), and REST POST. Individual data are represented in grey (dotted lines highlighting the two participants who did not feel illusions) and the median of all participants (*n* = 19) in black. p: *** ≤ 0.001, ** ≤ 0.01. (**B**) The heatmap (mean of all participants) for frequencies from 3 to 25 Hz. The blue color illustrates a desynchronization. (**C**) The scalp maps (of one representative participant) during the REST PRE (on the left), during LMV (on the middle), and the REST POST (on the right).

The EEG analysis at the EEG1 session revealed a significant desynchronization of the ERSP_MU_ over the 20-minute LMV, as indicated by a larg*e* effect size (χ^2^ = 51.338, p < 0.001, W = 0.450; see Fig. 3). Post-hoc analysis did not show a significant difference between REST PRE and REST POST (p = 0.56), or REST PRE and 19’ (*p* = 0.17). Significant modifications, however, were found between REST PRE and 1’ (*p* < 0.001), 5’ (*p* < 0.001), 10’ (*p* < 0.001), and 15’ (*p* = 0.003), as well as between 1’ and 15’, 19’, and REST POST (for all tests, *p* < 0.001).

### Correlation between subjective sensory estimation and ERSP_MU_

Inter-participant correlations were calculated between the pooled subjective scores provided by participants during the 20-minute LMV period and the recorded ERSP_REST_ during the session EEG1. The correlation did not reach significance (rho = 0.33, *p* = 0.17, supplemental Figure A.2 A). A repeated measures correlation analysis did not show any significant intra-participant correlation between the evolution of ERSP_REST_ and the subjective pooled illusions scores during EEG1 (*r* = 0.09, *p* = 0.44, supplemental Figure A.2B). This indicates that the subjective scores and the desynchronization patterns during LMV are independent.

### Neurophysiological and strength evaluations

**Fig. 4:**
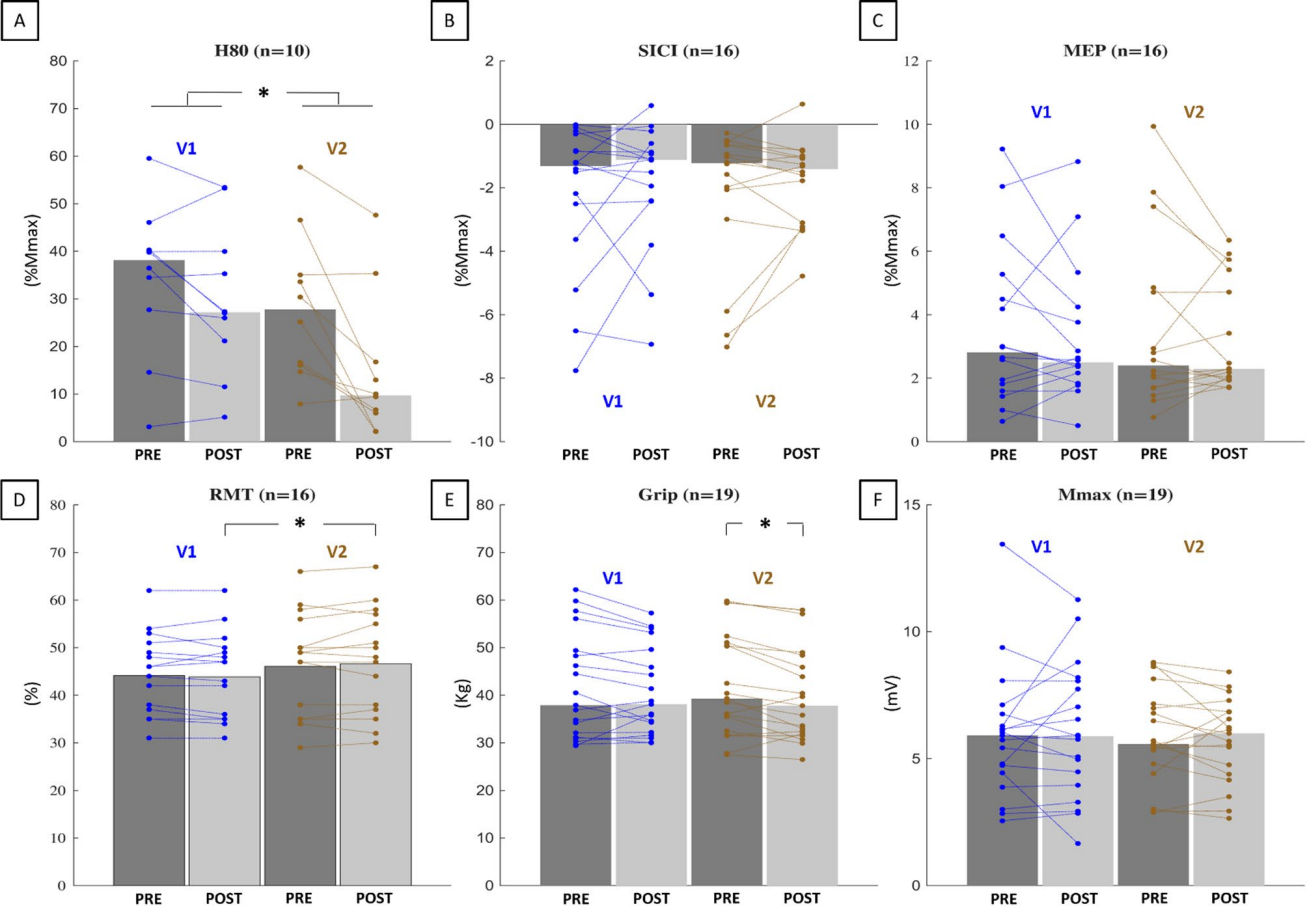
PRE and POST values for V1 (after 20-minute rest) and V2 (after 20-minute LMV). Dark grey bars represent PRE values, and light grey bars represent POST values. Bars without outlines show the group median, while bars with black outlines indicate the mean. Individual participant’s data are shown as lines and dots: blue for V1 and brown for V2. Statistical differences are indicated with p: * < 0.05. H80 = 80% of maximal H-reflex, SICI = short intracortical inhibition, MEP = Motor evoked potential, RMT = resting motor threshold, Mmax = maximal M-wave, %Mmax = expressed as a percentage of maximal M-wave.

**Fig. 5:**
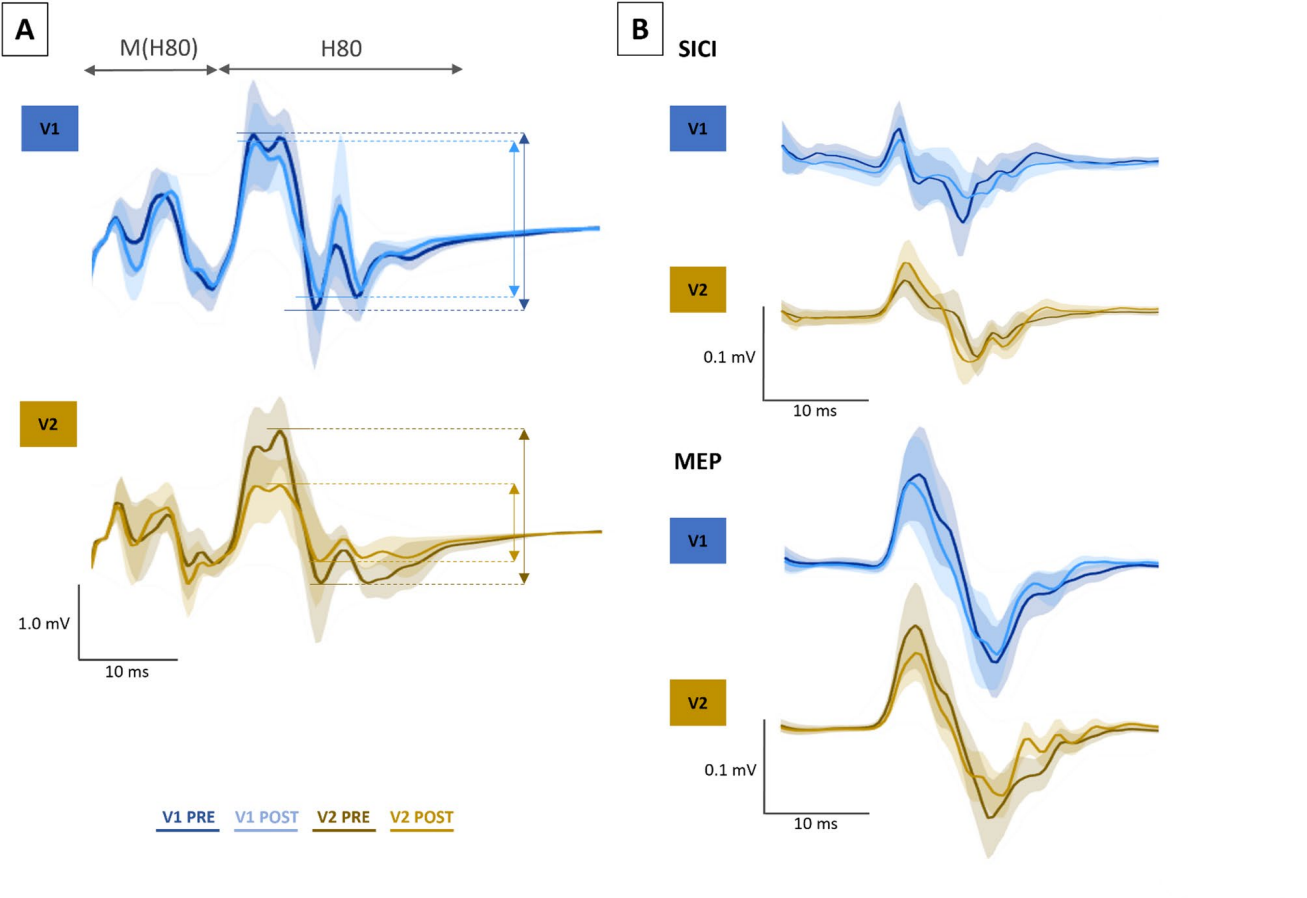
Mean traces of the H80 (**A**), SICI, and MEP (**B**) parameters during PRE and POST sessions in V1 (after 20 min rest) and V2 (after 20-minute LMV). Shaded areas represent the standard error. Blue curves indicate V1_PRE_ and V1_POST_, and brown curves indicate V2_PRE_ and V2_POST_.

Figure 4 shows the mean or median of all the parameters recorded in V1 and V2. For the H-reflex, 10 out of 19 participants exhibited a reflex in the FCR (at all stimulation intensities), with statistical analysis revealing a significant *Visits* effect (χ^2^ = 8.40, *p* = 0.038, W = 0.280; see Fig. 4A). Post-hoc comparisons did not reach significance (*p* ≥ 0.140). The Wilcoxon test, comparing V1_RATIO_ (median across participants: -8.2% [-28.8 ; 1.8]) and V2_RATIO_ (-52.3% [-69.7 ; -21.0]) revealed a significantly larger reduction of the H80 during V2 compared to V1 (*p* = 0.020, r_B_ = 0.818). A qualitative illustration of H-reflex reduction is depicted in Fig. 5A, which displays representative H-reflex waveforms from a typical participant in both V1 and V2.

The neurophysiological parameters presented in Fig. 4B, C, and Fig. 4F showed no significant effects, including SICI (χ^2^ = 0.375, *p* = 0.945, W = 0.008, see Fig. 5B), MEP (χ^2^ = 0.975, *p* = 0.807, W = 0.020 see Fig. 5B), and Mmax (χ^2^ = 0.537, *p* = 0.911, W = 0.009), and no V1_RATIO_ and V2_RATIO_ differences (*p* ≥ 595, r_B_ ≤ 0.162). For RMT (Fig. 4D), no Visits * PrePost interaction or *PrePost* effect was detected (F ≤ 1.215, *p* ≥ 0.288, ղ_p_^2^ ≤ 0.075). Nonetheless, a significant main effect of *Visits* was identified (F = 6.680, *p* = 0.021, ղ_p_^2^ = 0.308), indicating a slight increase in RMT in V2 in comparison to V1. It is worth noting that 3 out of 19 participants were excluded from all TMS-related measures due to either a lack of short intracortical inhibition or an excessively high resting motor threshold.

Regarding grip strength, a significant effect was found (χ^2^ = 8.952, *p* = 0.030, W = 0.157). Post-hoc analyses revealed a significant reduction in strength between V2_PRE_ and V2_POST_ (*p* = 0.039), while no other comparisons reached significance (*p* ≥ 0.510). However, the Wilcoxon test comparing V1_RATIO_ (median across participants: -3.8% [-7.0 ; 2.8] and V2_RATIO_ (-4.9% [-7.8 ; -2.6]) showed no significant difference in grip strength during V2 compared to V1 (*p* = 0.459, r_B_ = 0.205).

A detailed statistical analysis is presented in the supplemental Table A.2.

The acute effects observed in V2 following LMV, compared to the V1 control condition, align with previous findings in the literature. Specifically, we found a reduction in H-reflex amplitude^4,7^, no significant changes in MEP, SICI, or Mmax^7,17^, and a slight decrease in grip strength, though, not exceeding the reduction observed in the control condition^18^. These results confirm that our LMV application adheres to standard conditions.

### Chronic impact of LMV (inter-visit effects)

#### Assessment of protocol efficacy: subjective scales of sensory illusions

**Fig. 6:**
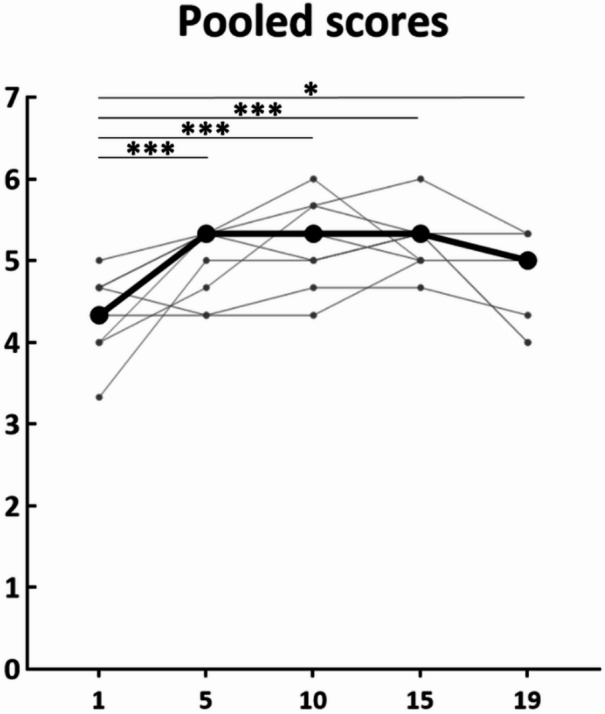
Evolution of the pooled scores (median of the 19 participants) at specific time points (1, 5, 10, 15, and 19 min) over the 9 vibratory sessions. In grey, the pooled scores by session (from V2 to EEG2), and in black the median of the 9 vibratory sessions. p: *** < 0.001 and * < 0.05.

Figure 6 shows, for the nine vibratory sessions (V2 to EEG2), the median pooled score of the three visual analogue scales used to subjectively rate the sensory illusions perceived by participants (n = 19). The majority of participants (n = 17) experienced sensory illusions, except for two participants who did not experience any throughout the entire protocol. The median sensations were rated at 3.7 [2.2 ; 5.8] for strength, 5.9 [2.4 ; 7.4] for continuity, and 4.4 [2.1 ; 5.7] for vividness over the 20 minutes across all LMV sessions. The repeated measure ANOVA revealed a significant *Time* effect (F = 34.819, p < 0.001, ղ_p_^2^ = 0.813, Fig. 6). Post-hoc analyses demonstrated significant differences between 1’ and 5’ (*p* < 0.001), 10’ (*p* < 0.001), 15’ (*p* < 0.001) and 19’ (*p* = 0.011), between 10’ and 19’ (*p* = 0.007), and between 15’ and 19’ (*p* = 0.020). A Friedman test did not reveal any significant *Session* effect (χ^2^ = 7.164, *p* = 0.519, W = 0.047), indicating that the illusions remain consistent across sessions.

### Assessment of protocol efficacy: EEG analysis during LMV

The analysis of the EEGs recorded during the EEG1 and EEG2 sessions revealed a large desynchronization of the ERSP_MU_ over the 20-minute LMV. Similar to the EEG1 session, a significant *Time* main effect (χ^2^ = 16.707, p = 0.002, W = 0.220) was observed in the EEG2 session. Post-hoc showed significant modifications between 1’ and 15’, 19’ (for all analyses, *p* ≤ 0.011), between 5’ and 19’ (*p* = 0.017), and between 10’ and 19’ (*p* = 0.010), confirming the strong desynchronization of the alpha band mainly during the first 10 min of both the first and last LMV sessions. No difference was found between the two sessions (*p* = 0.953, r_B_ = -0.021).

### Correlation between subjective sensory Estimation and ERSP_MU_

Similar to EEG1, no significant inter-participant correlations were found between the median subjective pooled scores reported during the 20-minute LMV period in EEG2 and the median ERSP_REST_ values recorded during the same session (rho = 0.04, *p* = 0.44). The repeated measures correlation analysis also revealed no significant intra-participant correlation between the evolution of ERSP_REST_ and subjective illusion pooled scores during EEG2 (*r* = -0.053, *p* = 0.647).

### Neurophysiological and strength evaluations

**Fig. 7:**
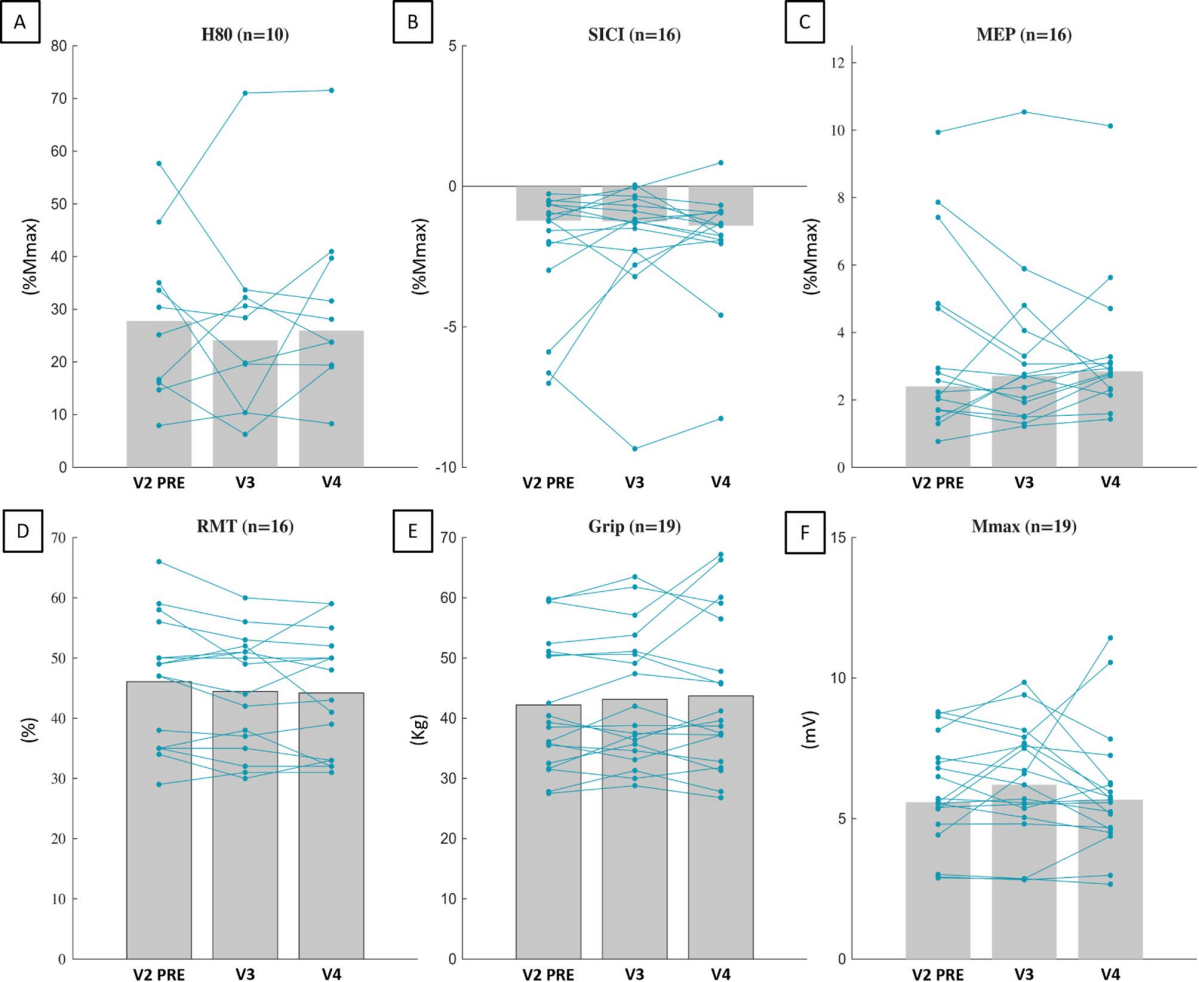
Evolution of the neurophysiological parameters for V2_PRE_ (the baseline), V3 (following the 9 LMV sessions), and V4 (five days after the end of the LMV). Grey bars represent group values, with medians shown by bars without outlines and means shown by bars with black outlines. Individual data are plotted in blue. H80 = 80% of maximal H-reflex, SICI = short intracortical inhibition, MEP = Motor evoked potential, RMT = resting motor threshold, Mmax = maximal M-wave, %Mmax = expressed as a percentage of maximal M-wave.

Figure 7 shows the group median or mean and individual values of all the evaluated parameters for V2_PRE_ (the baseline), V3 (after the nine sessions of LMV), and V4 (5 days after the end of the LMV protocol). None of the evaluated parameters exhibited chronic adaptation due to LMV-induced illusions. This was confirmed by the absence of *Visits* main effect (for all analyses, F ≤ 1.978, *p* ≥ 0.156, ղ_p_^2^ ≤ 0.153; χ^2^ ≤ 3.125, *p* ≥ 0.210, W ≤ 0.098). Statistical details are presented in supplemental Table A.4, and the evolution of M(H80) is presented in supplemental Figure A.3.

## Discussion

In this study, we investigated the neurophysiological and functional effects of nine sessions of LMV training designed to induce movement illusions. We assessed spinal, corticospinal, and intra-cortical excitabilities, as well as grip strength, of the vibrated FCR muscle in 19 healthy participants. Our results confirm that the LMV protocol induced movement illusions, as quantified by answers to a subjective questionnaire and by increased cortical activity (i.e., desynchronization of the Mu-band, ERSP_MU_ measured by EEG). At the acute level, our results aligned with the existing literature; that is a single LMV session significantly reduced spinal excitability^4,7^ without significantly changing muscle strength^18^, short intracortical inhibition or cortical excitability (MEP)^17^, when compared to the control condition. Following the nine sessions of LMV conducted across 11 days, no chronic modulations in intracortical or corticospinal excitabilities were observed, nor were there significant changes in grip strength. To our knowledge, this is the first study to examine the chronic effects of LMV on the upper limb under controlled conditions, with the aim of eliciting movement illusions.

In our study, both subjective (i.e., using visual analogue scales) and objective (i.e., EEG recording) measures were employed to evaluate movement illusions. Most participants (17 out of 19) reported experiencing illusions across the 9 LMV sessions, and all participants exhibited Mu band desynchronization over the sensorimotor cortex (C3), consistent with previous studies^14,27,28^. Our findings indicate that illusions can persist over extended durations, with the strongest sensations reported between 5 and 15 min of LMV. Correspondingly, cortical activity was significantly heightened during the first 15 min of LMV. This aligns with a previous study that assessed wrist illusions using the same subjective scales over 30 min of continuous LMV, observing sustained illusions throughout the session with peak scores at the beginning and a decrease toward the end^7^.

To our knowledge, this study is the first to repeatedly assess movement illusions using both methods. Interestingly, no significant correlation was found between these two variables, suggesting they may reflect distinct underlying processes. Although we might assume that subjective illusions are directly linked to cortical activity during LMV, our findings challenge this notion. One possible explanation is that the absence of a vibration condition without illusions limited our ability to establish a clearer relationship between these measures. Nonetheless, the significant effect of time observed for both variables indicates that meaningful fluctuations were captured, reinforcing the idea that they may represent independent processes.

Note that complementary correlation analyses were conducted to explore the relationship between movement illusions and various neurophysiological and strength measures, both acutely (see supplemental Table A.3) and chronically (see supplemental Table A.5). A strong inverse correlation was identified between subjective illusion ratings (across the mean scores of all scales: pooled scores) and the absence of a tonic vibratory reflex (TVR, see supplemental Table A.3). Similarly, a significant correlation was found between illusion ratings and grip strength, with higher pooled scores associated with less grip strength loss (see supplemental Table A.3). Both findings are consistent with the existing literature^3,18,20^. However, no such association was observed between ERPS_REST_ desynchronization and the absence of a TVR. It is notable that the EEG evaluation was conducted 1 day (EEG1) after the TVR measurement (V2), which may have influenced the observed results.

As previously mentioned, two participants did not experience any movement illusions throughout the experiment. To ensure the robustness of our findings, all correlations and analyses presented in the paper were re-evaluated after excluding these two subjects. The study’s outcomes remained unchanged.

Our protocol successfully replicated a well-documented effect of acute LMV on spinal excitability, namely a significant reduction in the H-reflex amplitude (-52.3% [-69.7 ; -21.0]) immediately following LMV^4,7,10^. This reduction in spinal loop excitability is consistent across studies, regardless of the specific LMV conditions employed^4^. No other significant acute effects of LMV were observed in our study. Consistent with prior research, we did not detect acute changes in RMT and Mmax, two parameters known to remain stable over time^7,19^.

Similarly, our findings align with studies employing LMV conditions similar to ours, which reported no acute effects on MEP amplitude^11,12,15^. Note, however, that a recent study has reported a MEP reduction following LMV with illusion^7^. It has been suggested that when measured by TMS, possible corticospinal increase may be masked by a reduction in motoneuronal excitability. Notably, some studies have reported acute increases in the MEP/CMEP ^7,11^ or MEP/TMEP^12^ ratios, indicating a transient rise in cortical excitability during the first 30 min following acute LMV. As our protocol did not evaluate motoneuronal excitability, we cannot confirm or refute these findings.

Our evaluation of short intracortical inhibition (SICI) revealed no acute effect of LMV. This aligns with findings from analogous LMV protocols that did not specifically control for movement illusions^15,17,51^. As MEP strongly influences SICI^52^, we calculated the SICI/MEP ratio to account for potential masked effects (see Supplemental Table A.2). Our analysis revealed no significant changes in this ratio, suggesting that variations in MEP amplitude do not confound the observed results. These observations challenge hypotheses suggesting that intracortical changes involving GABAergic circuits, particularly GABA_A_ pathways, are a mechanism for vibration-induced modulation of corticospinal excitability^4,30,51^.

Finally, we observed a slight reduction in strength post-vibrations (-4.9% [-7.8 ; -2.6]), which was not significantly greater than the reduction observed under resting conditions (-3.8% [-7.0 ; 2.8]). This finding corroborates those of some previous studies^4,53,54^ but differs from others^4,55^. Notably, two studies with a similar LMV protocol and controlling for movement illusions reported, for the first one, a significant acute reduction in the strength of the vibrated muscle (-11.8%)^7^, however, it lacked a control condition for comparison. The second study found no significant difference in maximal strength post-vibration condition with illusions, whereas a marked reduction in condition was observed without illusions but with TVR^18^.

No chronic adaptations to LMV were observed in our study. We found that repeated exposure to LMV induced no neurophysiological adaptations at the spinal level (i.e., H-reflex) in the vibrated muscle. This finding contributes to the limited literature, where studies have reported increased^31,32^ or unchanged^34^ H-reflex amplitudes following LMV training. These discrepancies may arise from variations in LMV parameters (i.e., 50 Hz for 2 weeks, 100 Hz for 8 weeks, and 80 Hz for 2 weeks, in the studies by Lapole et al. (2013)^31^, Souron et al. (2017)^34^ and ours, respectively), evaluation methods (Lapole (2013)^31^ did not adjust with M-wave (associated to the H-reflex) for equalization, Souron (2017)^34^ evaluated H-reflex during muscle contraction), duration of application (1 h per day in Lapole and Souron studies, and 20 min in ours), or the targeted segment (i.e., Achilles’ tendon for Lapole, tibialis anterior for Souron, wrist in our study).

As expected, the maximum M-wave and RMT remained stable across visits, consistent with previous findings indicating that these parameters are unaffected by time or LMV^19,32,34^. Moreover, we did not observe any training effects on corticospinal excitability (MEP), aligning with prior works that report no significant changes following LMV training^33,34^. However, the possibility of cortical adaptations cannot be entirely dismissed. A more comprehensive approach incorporating an evaluation of motoneuronal excitability, as described in the previous paragraph, could provide deeper insights.

Furthermore, no chronic impact of LMV on SICI was observed. This is the first study to measure the chronic effect of LMV on intracortical inhibition, as assessed by SICI. Our results align with those of a previous study, which reported no impact on silent period duration^34^. Still, they are in contrast with another work that found a reduction in this silent period^33^. The interpretation of these results is complicated by the fact that the methodology used to assess intracortical inhibition is influenced by confounding mechanisms that make it challenging to isolate and analyze^56^. To rule out masked effects, as was done in the previous acute part, we verified the SICI/MEP ratio, but no significant changes were detected (see supplemental Table A.4). Despite increased cortical activity in sensorimotor areas during LMV, our results did not reveal any lasting effects on cortical or intracortical mechanisms.

Lastly, our results showed no significant increase in strength over time, diverging from prior training studies that reported strength gains^31–34^. Although not statistically significant, our findings did indicate a slight tendency towards increased strength (V2_PRE_ to V3: 2.7 ± 8.0% and V2_PRE_ to V4: 3.3 ± 10.5%). This difference may be partly due to the shorter duration of our protocol (under 2 weeks), which may not be sufficient to induce neural adaptations and strength gain^4,57^, as demonstrated in studies with longer training durations. Also, our protocol did not elicit any acute effects of LMV on strength, suggesting that LMV applied under movement illusion conditions may influence strength differently than other LMV protocols, potentially leading to distinct training adaptations^18^. These findings highlight the need for further research to explore the chronic effects of LMV under controlled conditions, particularly those designed to specifically induce tonic vibratory reflex (TVR).

It is essential to consider that this type of strong sensory stimulation alone may not be sufficient to induce long-term plasticity without the integration of cognitive or motor tasks, as suggested by some studies using congruent action observation^30,58–60^. This warrants the need for further research to explore how cognitive engagement or task-specific activities could boost the effectiveness of LMV protocols aimed at promoting neuroplasticity.

LMV are widely used in clinical settings with numerous studies reporting their acute effects in chronic patients, such as a temporary reduction of spasticity. Several reviews offer valuable insights into their therapeutic applications^5,6,61–64^. However, the substantial variability in protocol parameters often leads to differing neurophysiological and clinical outcomes^64^. Accordingly, the underlying mechanisms of these effects remain insufficiently understood.

It is important to consider that studies conducted on healthy individuals using this type of training protocol may fail to elicit measurable chronic adaptations. By definition, these participants’ sensorimotor systems are already near a functional ceiling. Despite the relatively high LMV dose applied in our protocol (i.e., 20 min per day), this alone may not be sufficient to induce measurable neurophysiological or strength modulations. However, in pathological populations where the sensorimotor system is impaired, such interventions may hold significant therapeutic potential for improving function and compensating for neuromuscular deficits, pending rigorous evaluation within standardized clinical protocols.

Study limitations should be acknowledged. We selected the flexor carpi radialis (FCR) muscle due to its accessibility and relevance in assessing the upper limb extremity; however, this small muscle is known to produce relatively small MEP/Mmax ratios, often below 10% at rest^7^. This characteristic may contribute to higher variability in SICI measurements^46^. Indeed, our neurophysiological results exhibited variability, similar to other studies^17^, suggesting that a larger sample size might be necessary to strengthen the statistical power of our findings.

Only ten out of nineteen participants exhibited an H-reflex in the FCR, likely due to the methodological approach of measuring the reflex in a resting muscle, a condition that is suboptimal for eliciting this response^65^. However, this method was intentionally selected to assess excitabilities in a resting state, allowing us to investigate whether LMV could modulate the baseline neural activity in healthy participants.

Additionally, our results on grip strength should be interpreted with caution. Grip strength reflects the combined activity of multiple wrist and hand muscles, including moderate wrist extensors, not just the wrist flexors^66^. A more targeted assessment focusing exclusively on wrist flexors might have yielded more pronounced results. Existing literature suggests that LMV protocols inducing movement illusions may have a significant impact on the antagonist muscles involved in the perceived movement^7,26,67^. Exploring this phenomenon in future studies could provide valuable insights.

## Supplementary Information

Below is the link to the electronic supplementary material.

Supplementary Material 1

## Data Availability

The corresponding author can provide the datasets from this study upon reasonable request.
