# Supplementary material for: Acute and chronic effects of local muscle vibration training inducing illusions on wrist strength and neurophysiological measures
Source: Sci Rep. 2025 Nov 28;15:42773. doi: 10.1038/s41598-025-26915-z (PMC12663295; doi:10.1038/s41598-025-26915-z)
Supplement: Supplementary file 1 — Supplementary Material 1 [file 41598_2025_26915_MOESM1_ESM.pdf]

SUPPLEMENTALS MATERIAL

Table A.1: Mean number of stimulations analyzed per variable and participant at each visit, following the removal of outlier values, calculated with the IQR method. Results are presented as mean ± SD.

Measure	Number stimulations / session	$V1_{PRE}$	$V1_{POST}$	$V2_{PRE}$	$V2_{POST}$	V3	V4	Total loss (%)
**SICI** (n = 16)	15	13.6 ± 1.6	13.8 ± 1.2	14.1 ± 0.9	13.6 ± 1.5	13.8 ± 1.3	13.7 ± 1.2	8.4 ± 3.4
**MEP** (n = 16)	15	13.8 ± 1.2	14.0 ± 1.0	14.0 ± 1.4	13.8 ± 1.6	13.8 ± 1.1	13.8 ± 1.1	7.8 ± 3.0
**M(H80) & H80** (n = 10)	10	8.2 ± 1.5	8.8 ± 1.6	9.0 ± 0.8	8.7 ± 1.2	8.1 ± 1.4	8.7 ± 0.8	14.2 ± 4.6

SICI evaluation

Supplemental Figure 1 qualitatively illustrates Motor Evoked Potentials (MEPs) obtained from single and double (SICI) TMS stimulations. One-sample t-test comparing SICI values with the '0' zero value revealed significant differences for $V1_{PRE}$ ($t = -3.735$, $p = 0.002$), $V1_{POST}$ ($t = -3.732$, $p = 0.002$), and $V2_{PRE}$ ($t = -3.888$, $p = 0.001$). This finding indicates that the protocol used induced intracortical inhibition.

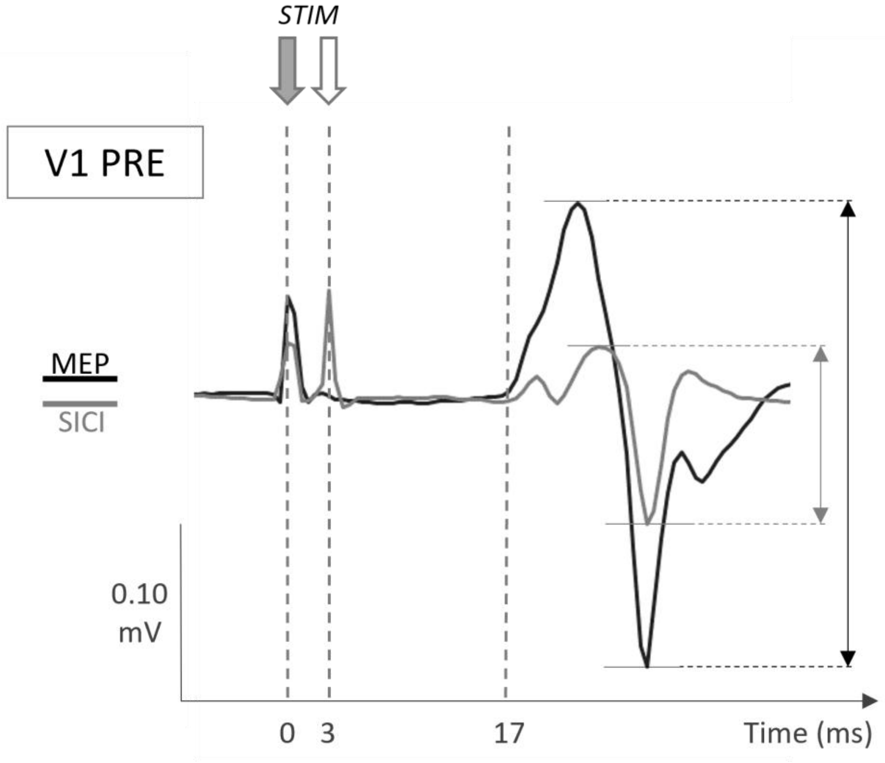

Supplemental Figure A.1: Motor evoked potential (MEP) after a single stimulation by TMS at 130% RMT (black), and after two stimulations at 80-130% RMT (SICI, in grey), are shown from one participant during $V1_{PRE}$.

Acute effects

Correlation between subjective sensory evaluation and EEG

No correlation was observed between the Event-Related Spectral Perturbation ($ERSP_{REST}$) and the subjective pooled scores (rho = 0.33, p = 0.17).

Repeated multiple correlation analysis did not show a significant intra-participant correlation between the evolution of $ERSP_{REST}$ and the subjective illusion pooled scores during EEG1 (r = 0.09, p = 0.44).

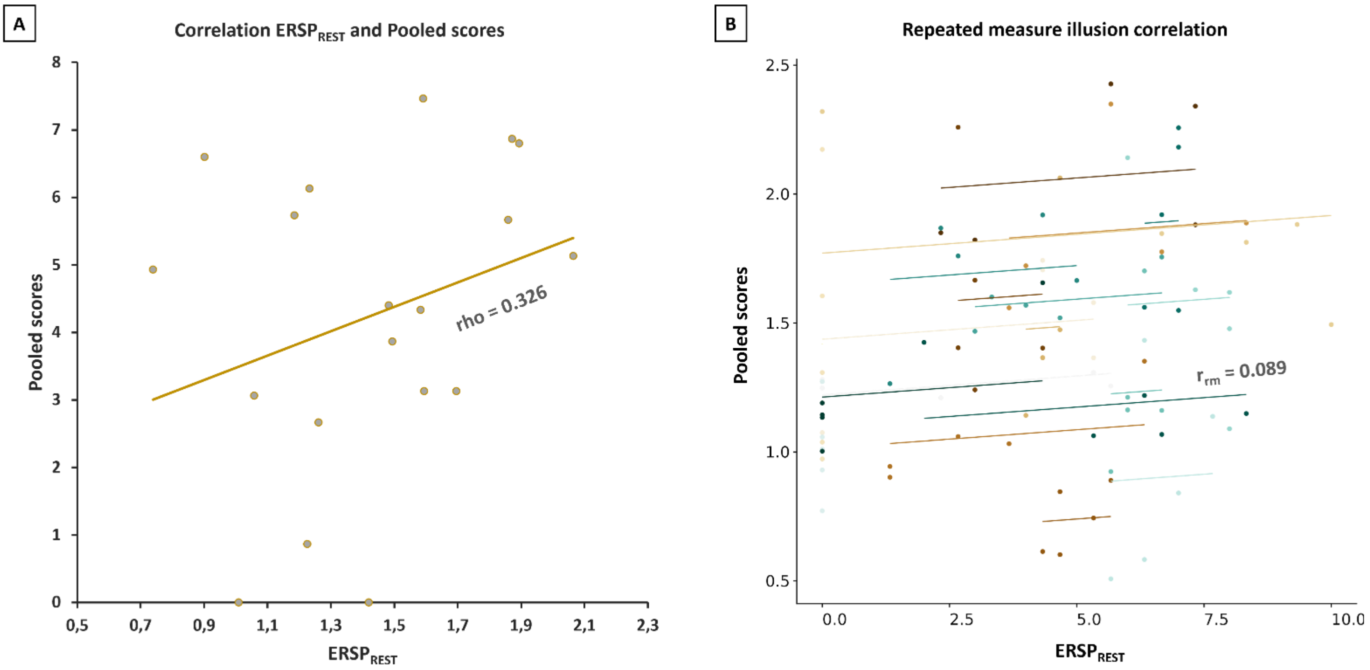

Supplemental Figure A.2: Figure A displays the inter-participant correlation between the mean subjective scores (Pooled) and the mean objective Event-Related Spectral Perturbation ($ERSP_{REST}$, EEG) across all participants (n = 19) during the second LMV session (EEG1). Figure B illustrates the intra-participant repeated measure correlation between the subjective pooled scores and $ERSP_{REST}$ for all participants (n = 19) across the entire 20-minute LMV of EEG1, at different time points (1', 5', 10', 15', and 19').

Supplemental Table A.2: Results for the acute impact of LMV. Two-factor repeated measures ANOVA, with *Visits* (V1, V2) and *PrePost* (PRE, POST) as factors, or Friedman test with a single factor, *Visits* ($V1_{PRE}$, $V1_{POST}$, $V2_{PRE}$, $V2_{POST}$), completed by a Wilcoxon test comparing $V1_{RATIO}$ and $V2_{RATIO}$. $p \leq 0.05$ is significant.

	Visits			*Post-Hoc*	*Wilcoxon*	
	χ^2	*p*	*W*		*p*	r_B
**SICI**	0.375	0.945	0.008		0.940	-0.029
**MEP**	0.975	0.807	0.020		0.597	-0.162
**SICI/MEP**	0.375	0.945	0.008		0.980	0.015
**H80**	8.400	**0.038**	0.280	$V1_{PRE}$-$V1_{POST}$: 0,891<br>$V2_{PRE}$-$V2_{POST}$: 0,532<br>$V1_{PRE}$-$V2_{PRE}$: 0,532<br>$V1_{POST}$-$V2_{POST}$: 0,140	**0.020**	0.818
**Mmax**	0.537	0.911	0.009		0.595	0.147
**Grip**	8.952	**0.030**	0.157	$V1_{PRE}$-$V1_{POST}$: 0,619<br>**$V2_{PRE}$-$V2_{POST}$: 0,039**<br>$V1_{PRE}$-$V2_{PRE}$: 0,510<br>$V1_{POST}$-$V2_{POST}$: 0,841	0.459	0.205

	Visits			*PrePost*			*Interaction*			*Post-Hoc*
	F	*p*	η_p^2	*F*	*P*	η_p^2	*F*	*p*	η_p^2	
**RMT**	6.680	**0.021**	0.308	0.394	0.539	0.026	1.215	0.288	0.075	**V1-V2 : 0.021**
**M(H80)**	2.271	0.166	0.201	0.003	0.958	<0.001	4.844	0.055	0.350	$V1_{PRE}$-$V1_{POST}$: 0.490<br>$V2_{PRE}$-$V2_{POST}$: 1.000<br>$V1_{PRE}$-$V2_{PRE}$: 0.430<br>$V1_{POST}$-$V2_{POST}$: 1.000

Supplemental Table A.3: Results of the linear Spearman's correlation between the mean subjective pooled scores recorded during V2, or the objective measure (mean $ERSP_{REST}$) obtained during EEG1, and the acute neurophysiological and strength modifications (i.e., $V2_{RATIO}$), across all participants (n = 19). Statistical significance was set at $p \leq 0.05$, with p-values adjusted using the Holm-Bonferroni correction.

	Grip		SICI		MEP		SICI/MEP		H80		TVR	
	rho	p	rho	p	rho	p	rho	p	rho	p	rho	p
**Pooled**	0.567	**0.055**	-0.213	1.000	-0.152	1.000	-0.043	0.875	-0.209	1.000	-0.602	**0.036**
**$ERSP_{REST}$**	0.346	0.882	-0.306	1.000	-0.200	0.912	-0.235	1.000	-0.297	1.000	-0.172	0.480

Chronic effects

Supplemental Table A.4: Results for the chronic impact of LMV training. Repeated measures ANOVA, or Friedman test, with a single factor, *Visits* ($V2_{PRE}$, V3, V4). $p < 0.05$ is significant.

	Visits		
	χ^2	*p*	*W*
**SICI**	0.875	0.646	0.027
**MEP**	3.125	0.210	0.098
**SICI/MEP**	0.875	0.646	0.027
**H80**	1.800	0.407	0.090
**Mmax**	2.000	0.368	0.053
	F	*p*	η_p^2
**RMT**	1.978	0.156	0.117
**Grip**	1.017	0.352	0.053
**M(H80)**	1.627	0.234	0.153

Supplemental Figure A.3: Evolution of the M(H80) in $V2_{PRE}$ (the baseline), V3 (after 9 LMV sessions), and V4 (5 days after LMV stopped). Individual data are represented in blue and the group mean is represented by the grey column. %Mmax = expressed as a percentage of maximal M-wave.

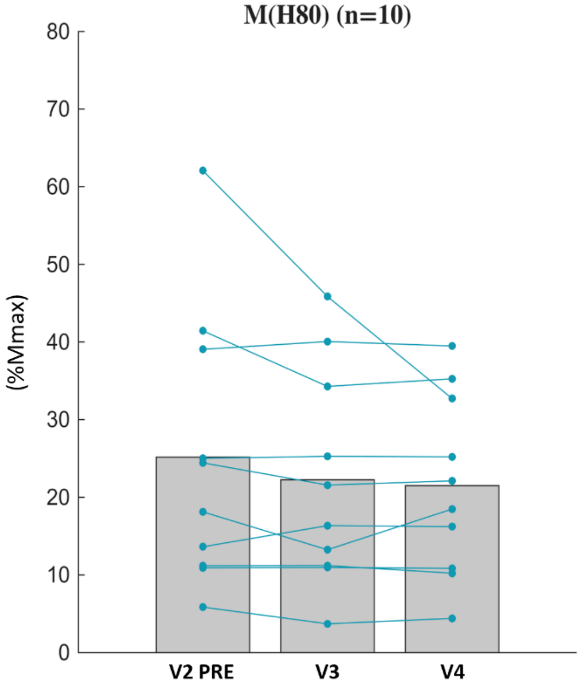

Supplemental Table A.5: Results of the linear Spearman's correlation between the mean subjective pooled scores recorded during V2 and V3, or the objective measure (mean $ERSP_{REST}$) obtained during EEG1 and EEG2, and the chronic neurophysiological and strength modifications (i.e., between $V2_{PRE}$ and V3), across all participants (n = 19). Statistical significance was set at $p \leq 0.05$, with p-values adjusted using the Holm-Bonferroni correction.

	**Grip**		**SICI**		**MEP**		**SICI/MEP**		**H80**	
	**rho**	**p**	**rho**	**p**	**rho**	**p**	**rho**	**p**	**rho**	**p**
**Pooled**	0.196	1.000	0.276	1.000	-0.071	0.794	0.416	0.545	0.243	1.000
**$ERSP_{REST}$**	0.111	1.000	0.079	1.000	-0.035	0.897	0.124	1.000	0.152	1.000
